# Retraction: The influence mechanism of mindfulness training on college students' psychological stress regulation: a mediation model with moderating effect

**DOI:** 10.3389/fpubh.2026.1901152

**Published:** 2026-06-09

**Authors:** Frontiers Editorial Office

The journal retracts the 26 February 2026 article cited above.

Following publication, concerns were raised regarding the validity of the data in the article. The authors failed to provide a satisfactory explanation during the investigation, which was conducted in accordance with Frontiers' policies.

This retraction was approved by the Chief Editors of Frontiers in Public Health and the Chief Executive Editor of Frontiers. The authors received a communication regarding the retraction and had a chance to respond. This communication has been recorded by the publisher.

